# Positive signs on physical examination are not always indications for endotracheal tube intubation in patients with facial burn

**DOI:** 10.1186/s12873-022-00594-9

**Published:** 2022-03-08

**Authors:** Ruo-Yi Huang, Szu-Jen Chen, Yen-Chang Hsiao, Ling-Wei Kuo, Chien-Hung Liao, Chi-Hsun Hsieh, Francesco Bajani, Chih-Yuan Fu

**Affiliations:** 1 Department of Trauma and Emergency Surgery, Chang Gung Memorial Hospital, Chang Gung University, Taoyuan, Taiwan; 2 Department of Plastic and Reconstructive Surgery, Chang Gung Memorial Hospital, Chang Gung University, Taoyuan, Taiwan

**Keywords:** Endotracheal tube, Facial burn, TBSA, Short of breath

## Abstract

**Background:**

After clinical evaluation in the emergency department (ED), facial burn patients are usually intubated to protect their airways. However, the possibility of unnecessary intubation or delayed intubation after admission exists. Objective criteria for the evaluation of inhalation injury and the need for airway protection in facial burn patients are needed.

**Methods:**

Facial burn patients between January 2013 and May 2016 were reviewed. Patients who were and were not intubated in the ED were compared. All the intubated patients received routine bronchoscopy and laboratory tests to evaluate whether they had inhalation injuries. The patients with and without confirmed inhalation injuries were compared. Multivariate logistic regression analysis was used to identify the independent risk factors for inhalation injuries in the facial burn patients. The reasons for intubation in the patients without inhalation injuries were also investigated.

**Results:**

During the study period, 121 patients were intubated in the ED among a total of 335 facial burn patients. Only 73 (60.3%) patients were later confirmed to have inhalation injuries on bronchoscopy. The comparison between the patients with and without inhalation injuries showed that shortness of breath (odds ratio = 3.376, *p* = 0.027) and high total body surface area (TBSA) (odds ratio = 1.038, *p* = 0.001) were independent risk factors for inhalation injury. Other physical signs (e.g., hoarseness, burned nostril hair, etc.), laboratory examinations and chest X-ray findings were not predictive of inhalation injury in facial burn patients. All the patients with a TBSA over 60% were intubated in the ED even if they did not have inhalation injuries.

**Conclusions:**

In the management of facial burn patients, positive signs on conventional physical examinations may not always be predictive of inhalation injury and the need for endotracheal tube intubation in the ED. More attention should be given to facial burn patients with shortness of breath and a high TBSA. Airway protection is needed in facial burn patients without inhalation injuries because of their associated injuries and treatments.

**Supplementary Information:**

The online version contains supplementary material available at 10.1186/s12873-022-00594-9.

## Background

According to current guidelines and treatment principles, endotracheal tube intubation is usually recommended in facial burn patients for the prevention of inhalation injury-related airway obstruction [1]. Patients with specific burn sites or specific findings on physical examinations, such as burned nasal hairs or carbonaceous debris in the mouth or sputum, may need early airway protection. Inhalation injury is a major cause of morbidity and mortality in patients with burn injury [2]. The immediately life-threatening consequence of inhalation injury is upper airway edema and further obstruction. It can be heralded by hoarseness, retraction, and stridor [3]. In addition to inhalation injury, intubation decisions in the emergency department (ED) for burn patients are sometimes made based on the burn type or site [4].

Practically, patients with positive findings on physical examination, which may indicate the need for airway protection, are not always intubated [5]. Some patients meet the current intubation criteria and undergo intubation in the ED; however, subsequent examinations confirm the absence of airway damage. These patients are soon extubated because there is no further need for airway protection. On the other hand, facial burn patients may need intubation due to other nonairway concerns. In other words, the diagnosis of inhalation injury remains a clinical judgment based on subjective evaluations. The probability of overresuscitation for airway injury exists in the management of burn patients. Therefore, it is important to develop an objective evaluation rather than relying on subjective “clinical judgment” to determine the need for airway protection in burn patients. Burn patients who need airway protection should be identified in the ED on the basis of clearer and more precise criteria. Such criteria would prevent unnecessary intubation.

In the current study, the characteristics of facial burn patients were studied. The role of conventional physical examinations in the decision regarding the need for airway protection was evaluated. We hypothesized that there are specific signs that can be evaluated in the ED to determine which burn patients need airway protection. With more precise intubation criteria, unnecessary intubation could be avoided, and delayed treatment could be minimized.

## Methods

Burn patients who presented at our ED were retrospectively reviewed in our trauma registry and medical records from January 2013 to June 2016. Patients with facial burns (International Classification of Diseases-9: 940.xx and 941.xx) were studied. Endotracheal tube intubation to ensure early airway protection was performed in patients with positive signs on physical examination (coughing, hoarseness, sore throat, shortness of breath or burn marks on nostril hairs, eyebrows, eyelids and hair), poor PaO_2_ or SaO_2_ on arterial blood gas (ABG) analysis or positive findings on chest X-ray (CXR), which could indicate inhalation injuries. In addition, patients may also be intubated based on the clinical judgment of the ED physicians. The exclusion criteria of the current study included age < 16 years, patients without facial burns or patients who arrived at our ED more than 72 h after the burn injury.

Our institution serves as a level I trauma center with 24/7 trauma surgeon and burn surgeon availability. Burn patients receive timely and comprehensive evaluations and treatment in our ED and burn center (ten beds for intensive care and ten beds in the general ward). All burn patients sent to our ED are managed according to a protocol based on the Advanced Burn Life Support (ABLS) guidelines [6]. After admission, inhalation injury is evaluated by subsequent examinations, including routine bronchoscopy and laboratory tests [7, 8]. Inhalation injuries are classified into three categories: (1) upper airway (above the glottis) injury, which is usually caused by thermal injury to the mouth, oropharynx or larynx; (2) lower airway (below the glottis), which is usually caused by the chemical or particulate constituents of smoke; and (3) asphyxiants, which is a process in which carbon monoxide or cyanide impairs oxygen delivery to the tissue [9]. Therefore, inhalation injury is confirmed if patients have positive bronchoscopic findings, such as bronchus/vocal cord edema, congestion, mucosal ulceration or necrosis or poor oxygenation on the laboratory test [7, 8, 10].

The severity of inhalation injury is evaluated per the abbreviated injury scale based on the bronchoscopy findings. (Supplementary Table 1) [11]. Grade 0 (absence of carbonaceous deposits, erythema, edema, bronchorrhea, or obstruction) indicates no inhalation injury, and grades 1 to 4 (1: mild injury, minor or patchy areas of erythema, carbonaceous deposits, bronchorrhea or bronchial obstruction; 2: moderate injury, moderate degree of erythema, carbonaceous deposits, bronchorrhea or bronchial obstruction; 3: severe injury, severe inflammation with friability, copious carbonaceous deposits, bronchorrhea, or obstruction; 4: massive injury, evidence of mucosal sloughing, necrosis, endoluminal obstruction) indicate inhalation injuries ranging from minor to severe. In the current study, general demographics (age, sex, type of burn, exposure to smoke or not), vital signs, Glasgow coma scale (GCS) score, laboratory examination results (arterial blood gas analysis and HbCO) and physical examination findings in the ED were recorded and analyzed. The total body surface area (TBSA) estimation and burn degree were obtained from the initial assessment by the specialist burn surgery team. The revised trauma score (RTS) was calculated to evaluate the condition of the trauma patients upon arrival at the ED [12].

First, patients who were and were not intubated were compared. Second, patients with and without definitive inhalation injury, as confirmed on subsequent bronchoscopy or laboratory tests, were compared. Then, statistically significant variables in the bivariate analysis were included in a multivariate logistic regression (MLR) model. Independent risk factors and the associated odds ratios for inhalation injury in facial burn patients were analyzed. Third, patients who did not undergo intubation in the ED but underwent intubation after admission were studied in detail. Finally, the relationship between the TBSA and the true need for airway protection was analyzed.

Statistical analysis was performed with Excel and SPSS™ (Statistical Package for the Social Sciences, Chicago, IL, USA). The numerical data are presented as the means ± standard deviations, and the nominal data are presented as numbers with percentages. Bivariate analyses were performed using Student’s t test and the chi-square test. A value of *p* < 0.05 was considered statistically significant. In the MLR model, a confidence interval (CI) not including or crossing 1.000 was considered statistically significant.

## Results

This retrospective study took place from January 2013 to June 2016 (42 months) and included an initial total of 1,004 burn patients, 335 of whom ultimately met the inclusion criteria. The mean age of the patients was 40.9 years, and 258 (77.0%) were male. The mean TBSA of these patients was 16.0%. The mean length of stay in the intensive care unit was 18.2 days, and the mean hospitalization duration was 20.3 days.

**Fig. 1:**
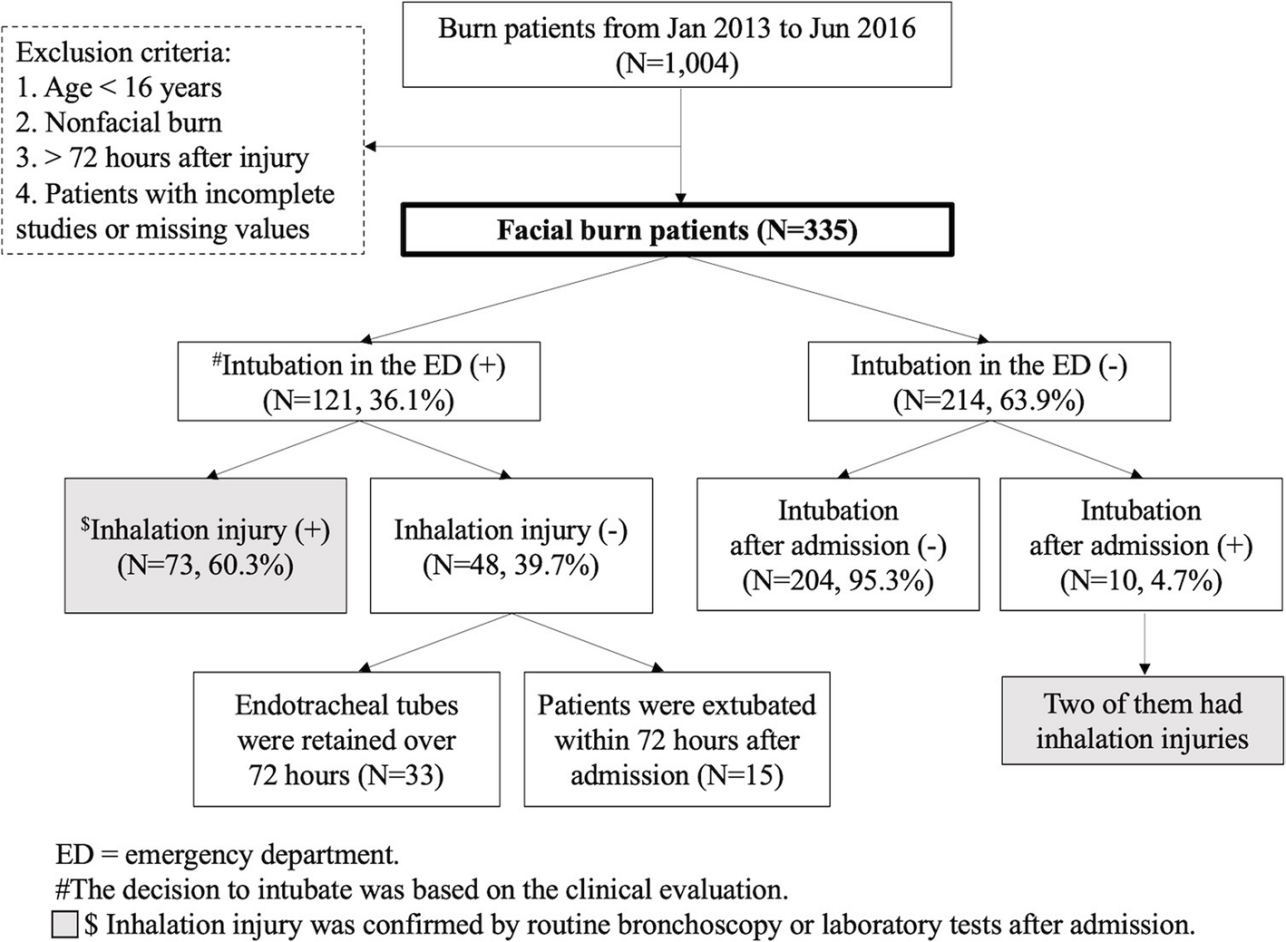
Study population, protocol and key numbers in the current study

The patient distribution and study protocol of the current study are shown in Fig. 1. In the ED, 121 patients (36.1%, 121/335) underwent endotracheal tube intubation based on the clinical judgment of ED physicians. Of these patients, 73 (60.3%, 73/121) had inhalation injuries that were confirmed on bronchoscopy or laboratory tests after admission, whereas 39.7% (48/121) of the intubated patients did not have inhalation injuries. In the patients without inhalation injuries who underwent intubation in the ED (*N* = 48), fifteen were extubated within three days after admission. In other words, the endotracheal tubes were retained for longer than three days in 33 patients because of other reasons for intubation (need for surgeries under general anesthesia, other associated injuries, etc.). On the other hand, among the patients who were not intubated in the ED, 10 (4.7%, 10/214) underwent intubation after admission. Two of them had inhalation injuries. Per the clinical judgment of ED physicians for primary intubations, the sensitivity and specificity of inhalation were 97.3% and 81.5%, respectively.

Table 1 compares the patients who were and were not intubated in the ED. The patients who were intubated in the ED had a significantly higher proportion of flame burns (77.7 vs. 46.7%, *p* < 0.001), lower GCSs (13.8 vs. 14.7, *p* < 0.001), higher TBSAs (27.9 vs. 9.8%, *p* < 0.001) and lower RTSs (7.6 vs. 7.8, *p* < 0.001) than the patients who were not intubated. In addition, the group of intubated patients had significantly higher proportions of patients with burned nostril hair (42.1 vs. 12.6%, *p* < 0.001), eye injuries (28.9 vs. 14.5%, *p* = 0.001), burned hair (25.6 vs. 11.5%, *p* = 0.001), hoarseness (7.4 vs. 0.9%, *p* = 0.001), shortness of breath (19.8 vs. 1.9%, *p* < 0.001), positive CXR (52.1 vs. 22.9%, *p* < 0.001) and exposure to smoke (19.8 vs. 5.1%, *p* < 0.001).

**Table 1:** Comparisons between facial burn patients who did and did not undergo intubation in the ED

Variables	Facial burn patients (*N* = 335)		*p*-value
	Intubation in the ED ( +) (*N* = 121)	Intubation in the ED (-) (*N* = 214)	
Demographics			
Age	38.0 ± 16.1	42.2 ± 18.9	0.064
Male	91 (75.2%)	167 (78.0%)	0.648^‡^
Types of burn (N, %)			< 0.001^‡^
Flame burn	94 (77.7%)	100 (46.7%)	
Scald burn	1 (0.8%)	49 (22.9%)	
Chemical burn	16 (13.2%)	47 (22.0%)	
Electrical burn	10 (8.3%)	18 (8.4%)	
TBSA (%)	27.9 ± 23.1	9.8 ± 5.5	< 0.001
Condition upon ED arrival			
SBP (mmHg)	154.1 ± 31.1	155.6 ± 28.6	0.652
Pulse (/minute)	99.8 ± 20.2	93.2 ± 64.4	0.219
RR (/minute)	20.9 ± 4.1	19.9 ± 8.6	0.516
Temperature (°C)	36.3 ± 0.7	36.4 ± 0.7	0.694
GCS	13.8 ± 2.6	14.7 ± 1.1	< 0.001
RTS	7.6 ± 0.6	7.8 ± 0.1	< 0.001
Laboratory examinations and imaging studies			
pH	7.3 ± 0.1	7.4 ± 0.1	< 0.001
PaCO_2_ (mmHg)	38.2 ± 13.0	36.5 ± 6.1	0.175
HCO_3_^−^ (mmol/L)	21.2 ± 3.8	23.4 ± 4.6	< 0.001
SaO_2_ (%)	90.5 ± 16.1	96.4 ± 61.5	0.315
HbCO (%)	3.4 ± 4.2	2.2 ± 1.7	0.008
Positive CXR (N, %)	63 (52.1%)	49 (22.9%)	< 0.001^‡^
Positive physical examination			
Nostril hair (N, %)	51 (42.1%)	27 (12.6%)	< 0.001^‡^
Eye (N, %)	35 (28.9%)	31 (14.5%)	0.001^‡^
Hair (N, %)	31 (25.6%)	25 (11.7%)	0.001^‡^
Cough (N, %)	6 (5.0%)	3 (1.4%)	0.053^‡^
Sore throat (N, %)	6 (5.0%)	3 (1.4%)	0.053^‡^
Hoarseness (N, %)	9 (7.4%)	2 (0.9%)	0.001^‡^
Dysphagia (N, %)	1 (0.8%)	0 (0.0%)	0.183^‡^
Shortness of breath (N, %)	24 (19.8%)	4 (1.9%)	< 0.001^‡^
Exposure to smoke (N, %)	24 (19.8%)	11 (5.1%)	< 0.001^‡^

*ED* Emergency department, *SBP* Systolic blood pressure, *RR* Respiratory rate, *CXR* Chest X-ray, *TBSA* Total body surface area, *RTS* Revised Trauma Score

Values are reported as the means ± SDs

Student’s t-test, ^‡^ chi-squared test

Table 2 compares patients with and without inhalation injuries as confirmed on bronchoscopy or laboratory tests after admission. Compared with the group of patients without inhalation injuries, the group of patients with inhalation injuries had a significantly lower GCS (13.8 vs. 14.5, *p* < 0.001), lower pH value (7.3 vs. 7.4, *p* < 0.001) and lower HCO_3_- level (20.5 vs. 23.1 mmol/L, *p* < 0.001) on arterial blood gas analysis and higher HbCO value (3.6 vs. 2.5%, *p* = 0.023), higher TBSA (31.5 vs. 12.1%, *p* < 0.001), lower RTS (7.5 vs. 7.8, *p* < 0.001) and larger percentages of patients with burned nostril hair (39.7 vs. 18.7%, *p* < 0.001), hoarseness (9.6 vs. 1.5%, *p* = 0.010), positive CXR (53.4 vs. 27.9%, *p* < 0.001) and exposure to smoke (19.2 vs. 8.0%, *p* = 0.011). However, the MLR analysis showed that only TBSA (odds ratio = 1.038, *p* = 0.001) and shortness of breath (odds ratio = 3.378, *p* = 0.027) were independent risk factors for inhalation injuries in facial burn patients (Table 3).

**Table 2:** Comparisons between facial burn patients with and without inhalation injuries

Variables	Facial burn patients (*N* = 335)		*p*-value
	Inhalation injury ( +) (*N* = 73)	Inhalation injury (-) (*N* = 262)	
Demographics			
Age	37.4 ± 13.4	41.6 ± 20.1	0.071
Male	51 (69.9%)	207 (79.0%)	0.841^‡^
Types of burn (N, %)			0.099^‡^
Flame burn 194	40 (54.8%)	154 (58.8%)	
Scald burn 50	11 (15.1%)	39 (14.9%)	
Chemical burn 63	14 (19.2%)	49 (18.7%)	
Electrical burn 28	6 (8.2%)	22 (8.4%)	
TBSA (%)	31.5 ± 18.1	12.1 ± 6.4	< 0.001
Condition upon ED arrival			
SBP (mmHg)	150.8 ± 39.6	156.2 ± 31.4	0.094
Pulse (/minute)	98.4 ± 19.5	94.8 ± 23.4	0.777
RR (/minute)	20.0 ± 1.4	20.3 ± 2.5	0.308
Temperature (°C)	36.4 ± 0.5	36.4 ± 0.6	1.000
GCS	13.8 ± 4.8	14.5 ± 2.6	< 0.001
RTS	7.5 ± 0.8	7.8 ± 0.2	< 0.001
Laboratory examinations and imaging studies			
pH	7.3 ± 0.4	7.4 ± 0.4	< 0.001
PaCO_2_ (mmHg)	39.2 ± 12.7	36.5 ± 7.0	0.047
HCO_3_^−^ (mmol/L)	20.5 ± 4.4	23.1 ± 4.2	< 0.001
SaO_2_ (%)	88.8 ± 12.5	95.5 ± 39.8	0.290
HbCO (%)	3.6 ± 2.1	2.5 ± 1.9	0.023
Positive physical examination			
Nostril hair (N, %)	29 (39.7%)	49 (18.7%)	< 0.001^‡^
Eye (N, %)	17 (23.3%)	49 (18.7%)	0.384^‡^
Hair (N, %)	17 (23.3%)	39 (14.9%)	0.089^‡^
Cough (N, %)	3 (4.1%)	6 (2.3%)	0.395^‡^
Sore throat (N, %)	2 (2.7%)	7 (2.7%)	0.975^‡^
Hoarseness (N, %)	7 (9.6%)	4 (1.5%)	0.010^‡^
Dysphagia (N, %)	0 (0.0%)	1 (0.4%)	1.000^‡^
Shortness of breath (N, %)	16 (21.9%)	12 (4.6%)	< 0.001^‡^
Positive CXR (N, %)	39 (53.4%)	73 (27.9%)	< 0.001^‡^
Exposure to smoke (N, %)	14 (19.2%)	21 (8.0%)	0.011^‡^

*ED* Emergency department, *SBP* Systolic blood pressure, *RR* Respiratory rate, *CXR* Chest X-ray, *TBSA* Total body surface area, *RTS* Revised Trauma Score

Values are reported as the means ± SDs

Student’s t-test, ^‡^ chi-squared test

**Table 3:** Multivariate logistic regression analysis identifying independent risk factors for inhalation injury in patients with facial burn

Variables	*p*-value^*^	Odds of inhalation injury	95% CI	
			Lower	Upper
GCS in ED	0.393	-	-	-
TBSA (%)	0.001	1.038	1.015	1.062
RTS	0.593	-	-	-
Nostril hair	0.208	-	-	-
Hoarseness	0.185	-	-	-
Shortness of breath	0.027	3.378	1.151	9.910
Positive CXR	0.351	-	-	-
Exposure to smoke	0.143	-	-	-
PH	0.559	-	-	-
PaCO_2_ (mmHg)	0.855	-	-	-
HCO_3_^−^ (mmol/L)	0.450	-	-	-
HbCO (%)	0.586	-	-	-

*GCS* Glasgow coma scale, *ED* Emergency department, *TBSA* Total body surface area, *RTS* Revised Trauma Score, *CXR* Chest X-ray, *CI* Confidence interval

^*^Multivariate logistic regression

**Fig. 2:**
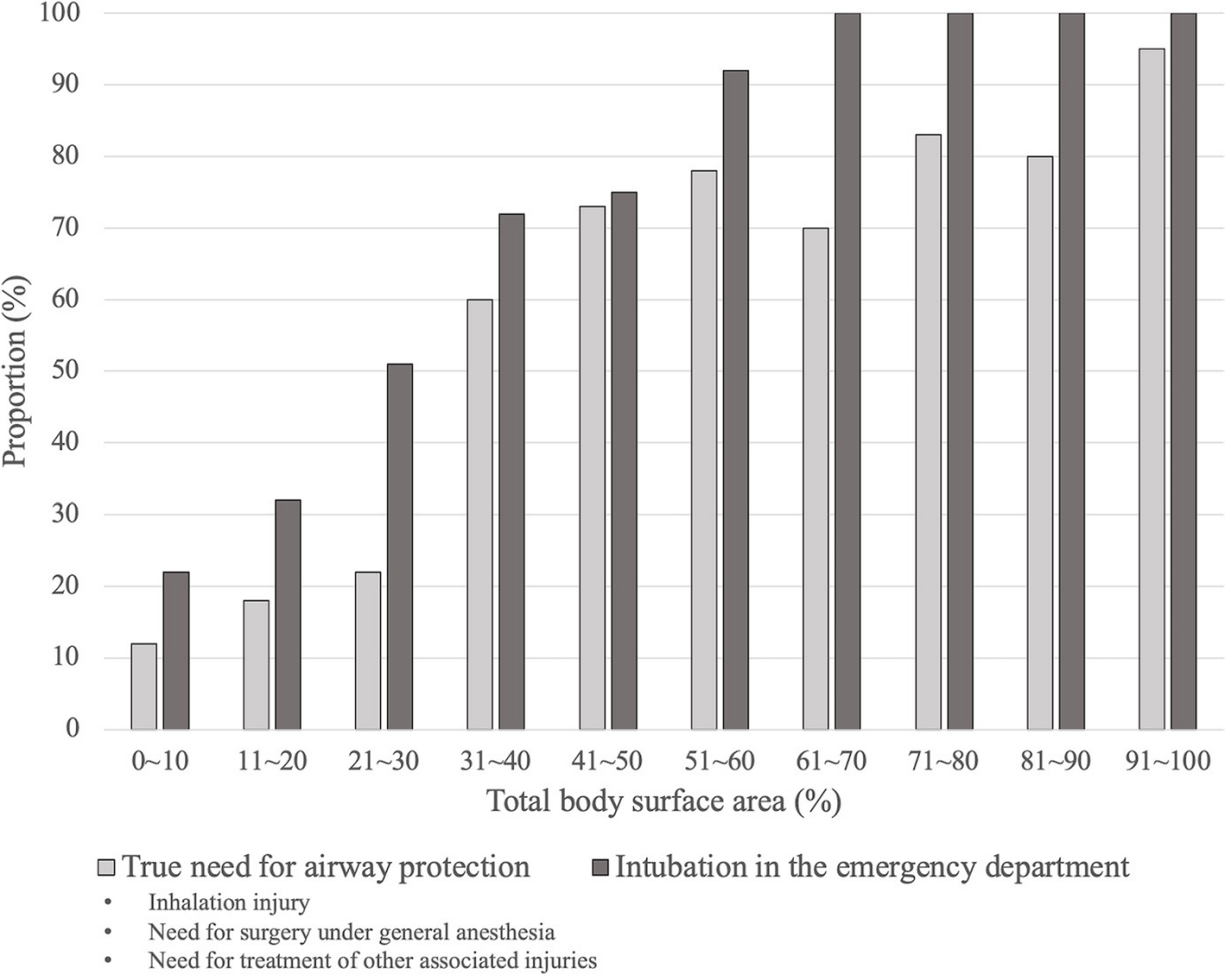
The relationship between the TBSA and proportion of patients who truly needed intubations and the relationship between the TBSA and the proportion of patients who were intubated in the ED

Figure 2 shows the relationships between the TBSA and the proportion of patients intubated in the ED and the proportion of patients who truly needed intubations (inhalation injury, need for surgery under general anesthesia, need for treatment of other injuries, etc.). With increasing TBSA, the proportions of both patients intubated in the ED and patients who truly needed intubations increased. In addition, the proportion of patients intubated in the ED was substantially higher than the proportion of patients who truly needed intubation in all categories of TBSA. All the patients with a TBSA over 60% were intubated in the ED regardless of whether they had inhalation injuries. The details of the patients who received endotracheal tube intubation after admission are listed in Table 4.

**Table 4:** Characteristics of patients who received delayed intubation after admission

Patient	Age	Sex	Reason for intubation	TBSA (%)	Intubation duration (days)
Patient 1	22	M	Surgery	18	2
Patient 2	37	M	Surgery	20	3
Patient 3	18	M	Surgery	27	5
Patient 4	25	M	Surgery	12	2
Patient 5	55	M	Pneumonia	50	19
Patient 6	31	F	Pneumonia	55	31
Patient 7	59	M	Shock	60	26
Patient 8	24	M	ARDS	60	28
Patient 9	65	M	Inhalation injury (Gr. 2)	35	15
Patient 10	42	M	Inhalation injury (Gr. 1)	20	11

*TBSA* Total body surface area, *ARDS* Acute respiratory distress syndrome

## Discussion

Traditionally, the diagnosis of inhalation injury has usually been based on a combination of clinical evaluations, such as patient history or physical examinations. Some physical findings have been considered indicators of a higher likelihood of laryngeal edema or inhalation injuries above the glottis and thus a greater need for intubation. A previous study demonstrated that stridor, shortness of breath, facial burns, singed nasal hairs, cough, soot in the oral cavity and history of being in an enclosed space with the fire should be strongly considered indicators for early intubation [13]. However, physical examinations are usually subjective evaluations. Diagnostic modalities such as laboratory examinations or chest X-ray may objectively contribute to the evaluation of inhalation injury [14, 15].

Similar to the findings of previous studies and current guidelines for facial burn management, in the current study, the patients who were intubated in the ED had significantly more positive signs on physical examinations or abnormal blood gas analysis results (Table 1). However, after undergoing bronchoscopy or laboratory tests as a definitive evaluation, 39.7% of the intubated patients were not found to have airway injuries (Fig. 1). The sensitivity of inhalation injury diagnosis was 97.3% based on the ED physician’s clinical judgment, but the specificity was only 81.5%. Furthermore, notably, the proportion of intubated patients was higher than the proportion with inhalation injuries, regardless of the TBSA (Fig. 2). These facts indicate that some airway protection procedures might be considered overresuscitation and that positive physical examinations are too sensitive to be used to make an intubation decision. Positive signs on physical examination may be indicators for intubation in the ED but may not correlate with the presence of a true need for intubation. In 2011, Mackie reported that a higher proportion of burn patients were treated with mechanical ventilators since 1997, whereas the severity of burn injury remained unchanged. A trend toward aggressive intubation for burn patients was observed [16].

Therefore, more objective evaluations of the indication for early intubation are needed. The MLR analysis in the current study revealed that most positive signs on physical examinations, laboratory examinations and CXR results could not be used to significantly predict inhalation injury (Tables 2 and 3). Shortness of breath is an easy-to-identify sign that is an independent risk factor for inhalation injury in facial burn patients. In addition, a high TBSA may also be independently predictive of inhalation injury. Every one percent increase in the TBSA was associated with a 1.038-fold increase in the odds of inhalation injury (*p* = 0.001, odds ratio = 1.038).

In the current study, positive correlations between TBSA and the odds of the need for intubation and intubation were both observed. All the patients with a high TBSA (> 60%) were intubated in the ED, even if they had no sign of inhalation injury. However, we observed that there were still approximately 20% of these high TBSA patients who had no true need for airway protection (inhalation injury on bronchoscopy, need for surgery or need for treatment of other injuries) and were extubated within three days after admission. It cannot be said that these intubations are “unnecessary”. We understand that the airway protection is a vital procedure for major burn patients during the primary evaluation and resuscitation. It is difficult to evaluate if the patient has true need for intubation or not within a short period in the ED. The aggressive resuscitation with airway protection is always considered. This implied that for severe burn patients (with a higher TBSA), inhalation injury is not the only indication for intubation. Steinvall, I and Liffner, G et al. reported that acute respiratory distress syndrome can develop in burn patients without inhalation injury due to an inflammatory process mediated by the effect of the burn, and this pathophysiology process is not associated with inhalation injury [17, 18]. Dries et al. also reported that critical burn patients may develop several lung injuries, such as sepsis, ventilator-induced lung injury or systemic inflammation, in addition to inhalation injury [19]. On the other hand, ten patients (8.3%, 10/121) underwent intubation after admission, not in the ED. Most of these patients (80%, 8/10) were intubated because they needed to undergo surgery under general anesthesia (four patients) or because they had complications of severe burn injuries (TBSA > 50%) (four patients). Only two patients had delayed symptoms of inhalation injury (shortness of breath or hoarseness) (Table 4). Among the patients who underwent intubation, 33 (27.3%, 33/121) patients did not have inhalation injuries (bronchoscopy grade = 0), although they remained intubated for longer than 3 days. Upper airway edema usually resolves within 2–3 days. These prolonged intubations implied that these patients were not intubated due to airway edema, a common complication of inhalation injury, and other reasons. Therefore, in addition to the management of inhalation injury, intubation still plays a significant role in stabilizing and treating other associated injuries. Airway management, as a part of resuscitation, should be considered for all burn-associated injuries, not only inhalation injuries.

The major limitations of this study are its retrospective nature and the small patient sample, which was obtained from a single institution. In addition, there were some patients with missing records of physical examinations and reasons for intubation. The aforementioned limitations notwithstanding, the results provide important information about the role of airway protection in the management of facial burn patients. A prospective study with a larger patient sample size should be designed to determine the accurate indications for endotracheal tube intubation in the ED.

## Conclusion

In the management of facial burn patients, conventional physical examinations may not always be predictive of inhalation injury and the need for endotracheal tube intubation in the ED. More attention should be given to facial burn patients with shortness of breath and a high TBSA. Airway protection may be needed in facial burn patients without inhalation injury because of their associated injuries and treatment.

## Supplementary Information

**Additional file 1.**

## Data Availability

Please contact author for data requests. (Fu, Chih-Yuan, E-mail: drfu5564@gmail.com ).

## Abbreviations

ED: Emergency department
ABG: Arterial blood gas
CXR: Chest X-ray
ATLS: Advanced Trauma Life Support
GCS: Glasgow coma scale
TBSA: Total body surface area
RTS: Revised trauma score
MLR: Multivariate logistic regression
SPSS: Statistical Package for the Social Sciences
CI: Confidence interval

## References

[1] Rue LW, Cioffi WG, Mason A, McManus W, Pruitt BA (1995). The risk of pneumonia in thermally injured patients requiring ventilatory support. J Burn Care Res.

[2] Mock C, Peck M, Peden M, Krug E (2008). A WHO plan for burn prevention and care.

[3] Traber DL, Hawkins HK, Enkhbaatar P, Cox RA, Schmalstieg FC, Zwischenberger JB (2007). The role of the bronchial circulation in the acute lung injury resulting from burn and smoke inhalation. Pulm Pharmacol Ther.

[4] Clark WR (1992). Smoke inhalation: diagnosis and treatment. World J Surg.

[5] Cancio LC (2009). Airway management and smoke inhalation injury in the burn patient. Clin Plast Surg.

[6] American Burn Association Advisory Committee (2018). American Burn Association provider manual.

[7] Ziegler B, Hundeshagen G, Uhlmann L, Will Marks P, Horter J, Kneser U, Hirche C. Impact of diagnostic bronchoscopy in burned adults with suspected inhalation injury. Burns. 2019;45(6):1275–82. 10.1016/j.burns.2019.07.011. Epub 2019 Aug 3. 10.1016/j.burns.2019.07.011 31383606

[8] Walker PF, Buehner MF, Wood LA, Boyer NL, Driscoll IR, Lundy JB (2015). Diagnosis and management of inhalation injury: an updated review. Crit Care.

[9] McCall JE, Cahill TJ (2005). Respiratory care of the burn patient. J Burn Care Rehabil.

[10] Bai C, Huang H, Yao X, Zhu S, Li B, Hang J (2013). Application of flexible bronchoscopy in inhalation lung injury. Diagn Pathol.

[11] Albright JM, Davis CS, Bird MD, Ramirez L, Kim H, Burnham EL (2012). The acute pulmonary inflammatory response to the graded severity of smoke inhalation injury. Crit Care Med.

[12] Champion HR, Sacco WJ, Copes WS, Gann DS, Gennarelli TA, Flanagan ME (1989). A revision of the Trauma Score. J Trauma.

[13] Vivó C, Galeiras R, del Caz MD (2016). Initial evaluation and management of the critical burn patient. Med Intensiva.

[14] Megahed MA, Ghareeb F, Kishk T, El-Barah A, Abou-Gereda H, El-Fol H (2008). Blood gases as an indicator of inhalation injury and prognosis in burn patients. Ann Burns Fire Disasters.

[15] Onishi S, Osuka A, Kuroki Y, Ueyama M (2017). Indications of early intubation for patients with inhalation injury. Acute Med Surg.

[16] Mackie DP, van Dehn F, Knape P, Breederveld RS, Boer C (2011). Increase in early mechanical ventilation of burn patients: an effect of current emergency trauma management?. J Trauma.

[17] Steinvall I, Bak Z, Sjoberg F (2008). Acute respiratory distress syndrome is as important as inhalation injury for the development of respiratory dysfunction in major burns. Burns.

[18] Liffner G, Bak Z, Reske A, Sjöberg F (2005). Inhalation injury assessed by score does not contribute to the development of acute respiratory distress syndrome in burn victims. Burns.

[19] Dries DJ, Endorf FW (2013). Inhalation injury: epidemiology, pathology, treatment strategies. Scand J Trauma Resusc Emerg Med.

