# Supplementary material for: Positive signs on physical examination are not always indications for endotracheal tube intubation in patients with facial burn
Source: BMC Emerg Med. 2022 Mar 8;22:36. doi: 10.1186/s12873-022-00594-9 (PMC8903723; doi:10.1186/s12873-022-00594-9)
Supplement: Supplementary file 1 — Additional file 1. [file 12873_2022_594_MOESM1_ESM.docx]

Supplementary Table. The abbreviated injury score grading scale for inhalation injury on bronchoscopy

| Grade | Class | Description |
| --- | --- | --- |
| 0 | No injury | Absence of carbonaceous deposits, erythema, edema, bronchorrhea, or obstruction |
| 1 | Mild injury | Minor or patchy areas of erythema, carbonaceous deposits, bronchorrhea or bronchial obstruction |
| 2 | Moderate injury | Moderate degree of erythema, carbonaceous deposits, bronchorrhea or bronchial obstruction |
| 3 | Severe injury | Severe inflammation with friability, copious carbonaceous deposits, bronchorrhea, or obstruction |
| 4 | Massive injury | Evidence of mucosal sloughing, necrosis, endoluminal obstruction |
